# The effect of early weight‐bearing and later weight‐bearing rehabilitation interventions on outcomes after ankle fracture surgery: A systematic review and meta‐analysis of randomised controlled trials

**DOI:** 10.1002/jfa2.12011

**Published:** 2024-04-18

**Authors:** Bocheng Chen, Ziyan Ye, Jiaxin Wu, Guoxiang Wang, Tiancheng Yu

**Affiliations:** ^1^ Physical Education and Sports School of Soochow University Soochow University Suzhou Jiangsu Province China

**Keywords:** ankle fracture, early weight bearing, post‐operative rehabilitation

## Abstract

**Objective:**

This systematic review aimed to analyse the effect of early weight bearing versus late weight bearing on rehabilitation outcomes after ankle fractures, which primarily include ankle function scores, time to return to work/daily life and complication rates.

**Methods:**

The China National Knowledge Infrastructure, Wanfang Data Knowledge Service Platform, China Science and Technology Journal, Web of Science, PubMed, Embase and Cochrane Library databases were searched. The focus was on identifying randomised controlled trials centred on early weight‐bearing interventions for post‐operative ankle fracture rehabilitation. All databases were searched for eligible studies published within the period from database inception to 20 June 2023. The eligible studies were screened according to the inclusion criteria. Study quality was evaluated using the methodology recommended by the Cochrane Handbook for the Systematic Evaluation of Interventions. Two authors independently performed the literature search and data extraction. Eligible studies were subjected to meta‐analyses using Review Manager 5.3. Based on the time points at which post‐operative ankle function was reported in the studies included in this paper, we decided to perform a meta‐analysis of ankle function scores at 6 weeks post‐operatively, 12 weeks post‐operatively, 24–26 weeks post‐operatively and 1 year post‐operatively.

**Results:**

A total of 11 papers, comprising 862 patients, were included. Meta‐analysis indicated that patients receiving early weight‐bearing interventions, which referred to weight‐bearing for 6 weeks post‐operatively, experienced enhancements in ankle function scores (Olerud–Molander score, AOFAS score or Baird–Jackson score) at various post‐operative milestones: 6 weeks (SMD = 0.69, 95% CI: 0.49–0.88 and *p* < 0.01), 12 weeks (SMD = 0.57, 95% CI: 0.22–0.92 and *p* < 0.01) and the 24–26 weeks range (SMD = 0.52, 95% CI: 0.20–0.85 and *p* < 0.01). The results of subgroup analyses revealed that the effects of early weight‐bearing interventions were influenced by ankle range‐of‐motion exercises. Additionally, early weight bearing allows patients to return to daily life and work earlier, which was evaluated by time when they resumed their preinjury activities (MD = −2.74, 95% CI: −3.46 to −2.02 and *p* < 0.01), with no distinct elevation in the incidence of complications (RR = 1.49, 95% CI: 0.85–2.61 and *p* > 0.05).

**Conclusion:**

The results showed that early weight bearing is effective in improving ankle function among post‐operative ankle fracture patients and allows patients to return to daily life earlier. Significantly, the safety profile of early weight bearing remains favourable, with no higher risk of complications than late weight bearing.

## 1 INTRODUCTION

Ankle fractures are the most common type of lower limb fracture and one of the most common types of fracture worldwide [1]. Such fractures can result in muscular contractures around the ankle joint, reducing ankle mobility and influencing an individual's capacity for daily activities [2]. Displaced or dislocated unstable ankle fractures are one of the most common types of ankle fractures. As per the Arbeitsgemeinschaft für Osteosynthesefragen/Orthopaedic Trauma Association (AO/OTA) classification [3], operative treatment is usually required for these types of fractures [4], and the surgical protocol is generally open reduction and external fixation, which is currently the most commonly applied surgical protocol. Nevertheless, while many patients have good surgical results, the absence of effective post‐operative weight‐bearing rehabilitation exercises gives rise to a range of issues, including soft tissue contractures and ankle joint osteoporosis, which ultimately result in poor recovery of foot function [5, 6].

Early weight bearing (EWB) is the practice of having a patient stand within 6 weeks of ankle surgery, with the operated ankle joint stressed by partial or full weight bearing [7, 8, 9]. It has been shown that EWB can provide a number of benefits to patients, including earlier pressure stimulation of the affected ankle joint, which promotes fracture healing and the recovery of ankle function [6, 10]. Among manual labourers who sustained ankle fractures, Cunningham et al. [11] found that ankle function scores and mobility scores were significantly higher in patients with EWB than in those with no weight bearing at the sixth week after surgery. A study by Smeeing et al. [2] also indicated that the recovery of ankle function (including ankle joint morphology, joint mobility and walking distance) in patients with EWB was superior to that in patients with a conventional post‐operative ankle rehabilitation protocol.

Unfortunately, late weight bearing (LWB) remains the current dominant rehabilitation protocol after ORIF [12, 13]. LWB is defined as providing ankle weight‐bearing interventions at 6 weeks post‐operatively or no weight bearing. LWB, as the traditional post‐operative fracture rehabilitation program, has a high level of safety and is effective in avoiding the loss of reduction or fixation failure after surgery, thus reducing the risk of secondary surgery. In contrast, LWB may affect the recovery of patients' ankle function [14, 15], and it may bring a heavier financial burden to patients, as LWB requires a longer rehabilitation time [16, 17, 18]. Therefore, researchers have recently begun to focus on the application of EWB in post‐operative ankle fracture rehabilitation programmes, considering that EWB can accelerate the speed of post‐operative ankle function recovery compared to LWB and enable patients to return to work/daily life earlier [6, 10]. There is still controversy, however, about the effect of EWB on the post‐operative rehabilitation of patients with ankle fractures [19], including whether EWB can improve mid‐ and long‐term ankle function and shorten the time for patients to return to work/life. Sharma et al. [20], for example, showed that post‐operative ankle fracture patients had better foot and ankle function within 12 weeks of receiving an EWB intervention than those who received an LWB intervention and were able to return to work/daily life earlier. On the other hand, in a meta‐analysis by Khojaly et al. [21], a significant difference in the functional recovery of the foot and the ankle between patients receiving EWB and LWB interventions was only present within 6 weeks, and there was no significant difference in the time to return to work/daily life between the EWB and LWB groups. Therefore, the issue of EWB's midterm and long‐term effects in post‐operative rehabilitation of ankle fractures (comparison of ankle function scores at 12 weeks post‐operatively and beyond) still needs further insights. Meanwhile, whether EWB helps patients to return to daily life/work earlier also needs further confirmation.

In light of these controversies, in this study, a meta‐analysis was used to compare the differences between EWB and LWB in terms of ankle function scores after ankle fracture surgery, especially at the 12th week after surgery and beyond and the time to return to work/daily life. In addition, although the safety of EWB has not been questioned in any meta‐analyses related to EWB and post‐operative rehabilitation of ankle fractures in recent years [20, 21, 22], considering that there are still some studies that reported a higher rate of complications of EWB than LWB [15], the present study also compared the rate of complications between patients receiving EWB and LWB interventions to further confirm the safety of EWB.

## 2 METHODS

Meta‐analyses were performed in accordance with the specifications of the Preferred Reporting Items for Systematic Reviews and Meta‐Analyses (PRISMA) statement [23]. The protocol was prospectively registered with the International Prospective Register of Systematic Reviews under the number CRD42023422525.

### 2.1 Retrieval strategy

The China National Knowledge Infrastructure (CNKI), Wanfang Data Knowledge Service Platform, China Science and Technology Journal (CQVIP), Web of Science, PubMed, Embase and Cochrane Library databases were searched, and the search period was from database inception to 20 June 2023. The search method included MeSH terms with added keywords, and the search terms were ankle fractures, lateral malleolar fractures, trimalleolar ankle fractures, medial malleolar fractures, lateral malleolar fractures, posterior malleolar fractures, bimalleolar ankle fractures, post‐operative management, early weight bearing, early loading, and immediate weight bearing. The retrieved documents were imported into Zotero software. The Chinese databases were searched until 20 June 2023 in line with the English databases. The studies retrieved were from three journals. After the search was completed and duplicates were removed, two researchers (BC.C and ZY.Y) independently screened the studies according to the inclusion and exclusion criteria. The studies were initially screened by reading the title and abstract, downloading the full text of the screened studies, reading through the full text and extracting information. The two researchers (BC.C and ZY.Y) compared the extracted information, and if there was any disagreement, a third researcher (JX.W) was consulted to discuss and decide whether the study should be included. Three studies from Chinese databases and eight from English studies were included.

### 2.2 Literature inclusion and exclusion criteria

#### 2.2.1 Inclusion criteria

(i) The study participants were patients with ankle fractures who underwent surgical treatment, with no restriction on the type of fracture or fixation.
(ii) The intervention involved weight bearing while standing on the affected ankle for a duration of 6 weeks post‐operatively. This encompassed both partial weight bearing with toe‐only support and complete weight bearing with full plantar contact.
(iii) The control group started weight bearing after 6 weeks post‐operatively or did not bear weight post‐operatively.
(iv) The outcome indicators included at least one of the scales associated with ankle function scores (only including the Olerud–Molander score, the AOFAS score and the Baird–Jackson score). These three scores are all assessed from three aspects of ankle: pain, ankle mobility and the ability to perform movements such as walking, running, jumping and ascending steps. Each of the scales ranges from 0 to 100. Additional outcome indicators were the number of complications and the patients' time to return to work/daily life,
(v) The study design was a randomised controlled trial (RCT).

#### 2.2.2 Exclusion criteria

(i) Studies without available full texts;
(ii) Studies wherein the experimental outcomes either remained unmeasured or could not be transformed into the mean ± standard deviation ($\bar{x}$ ± *s*);
(iii) Conference papers and dissertations;
(iv) Studies concerning subjects with concurrent conditions that could impede the adoption of early weight‐bearing interventions (e.g., cognitive impairment and neurological disorders).

### 2.3 Data extraction

The extraction of crucial data from eligible studies was conducted independently by two researchers (BC.C and ZY.Y). The extracted information encompassed the following:

(i) study characteristics, such as the author and publication date;
(ii) patient characteristics, including the overall sample size, surgical fixation approach and the number of patients in the experimental and control groups, as well as the age and sex distribution;
(iii) intervention characteristics, including the start time of weight bearing, the extent of the weight‐bearing load, the protective measures employed and whether active/passive ankle activities were integrated; and
(iv) outcome measures, including ankle function assessments (encompassing the Olerud–Molander score, AOFAS score and Baird–Jackson score), the incidence of complications and the time to return to work/daily life for patients, which was measured by the time when they resumed their preinjury activities [12].

### 2.4 Risk of bias evaluation

The risk of bias for all included studies was assessed according to the Cochrane 5.1 Handbook [24], including six distinct aspects: random sequence generation, allocation concealment, participant and outcome blinding, incomplete follow‐up, selective reporting and other biases. A low risk of bias was considered if a criterion was met, a high risk of bias was considered if a criterion was not met and an indeterminate risk of bias was considered if the criterion was not mentioned in the text with a note regarding the reason. The risk of bias of each study was evaluated by two researchers (BC.C and ZY.Y) independently, and in case of disagreement, a third researcher (JX.W) was consulted to discuss and reach a unanimous opinion.

### 2.5 Data analysis

This article analysed three outcome indicators (i.e., post‐operative ankle functional recovery, the time to return to work/daily life and the complication rate) from the included studies. All the studies included in this study reported patients' ankle function recovery at the following time points, so a meta‐analysis was performed at these time points: (i) six weeks post‐operatively; (ii) 12 weeks post‐operatively; (iii) 24–26 weeks post‐operatively; and (iv) one year post‐operatively [20, 21]. Data reported at the same time points were analysed separately by meta‐analysis. Studies that only reported median, maximum and minimum values estimated the mean and standard deviation using the method proposed by McGrath et al. [25].

Meta‐analysis was performed using Review Manager 5.3. A forest plot was used to represent the results of the study. Since the scales used to evaluate post‐operative ankle function differed among the different studies, the standardised mean difference (SMD) was used to pool ankle function scores, while the mean difference (MD) was used to pool the time to return to work/daily life. SMD is used in studies where measurements are made with different scales or units, allowing results to be compared between studies. Typically SMD = 0 indicates no difference between the two groups, SMD <0.2 is considered a small effect size, SMD between 0.2 and 0.5 is considered a medium effect size and SMD >0.5 is considered a large effect size. We used risk ratios (RRs) to synthesise dichotomous outcome data. Statistical heterogeneity was tested using a random‐effects model, which was selected for meta‐analysis if the *I* ^2^ statistic was >50% and *p* was <0.05, and a fixed‐effects model was used if the *I* ^2^ statistic was <50% and *p* was >0.05 [26]. Subgroup analyses or sensitivity assessments were used to judge sources of heterogeneity.

## 3 RESULTS

### 3.1 Search results

**FIGURE 1:**
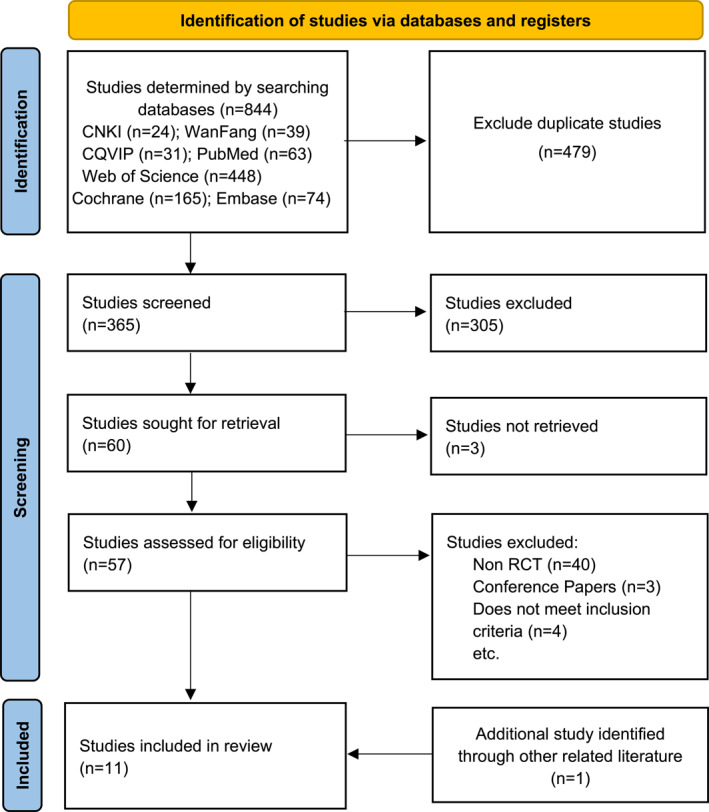
Inclusion screening studies process and results.

The process of the study search and screening is detailed in Figure 1. In June 2023, a total of 844 records were initially retrieved. These included 24 studies from the China National Knowledge Infrastructure database, 39 from the Wanfang Data Knowledge Service Platform, 31 from the China Science and Technology Journal Database, 63 from PubMed, 448 from the Web of Science, 74 from Embase and 165 from the Cochrane Library. The studies were imported into Zotero software, and 365 studies were obtained after eliminating duplicates. The titles and abstracts were read to exclude reviews, conference papers, studies and interventions that did not meet the inclusion criteria for this study, and 60 studies were screened by reading the full text. Based on the inclusion and exclusion criteria, a total of 10 of these articles were included in this study. The references of relevant studies were also manually searched, and one document that met the inclusion criteria was included. Consequently, 11 randomised controlled trials were ultimately included in this study [2, 5, 12, 15, 27, 28, 29, 30, 31, 32, 33]. The search process is shown in Figure 1.

### 3.2 Characteristics of the included studies

All 11 studies included in the meta‐analysis were randomised controlled trials. The study by Smeeing et al. [2] was a two‐arm study with two experimental groups and one control group, all of which met the inclusion criteria for this study. The means and standard deviations of the two experimental groups were combined in this paper using the algorithm recommended in the Cochrane Handbook. Thus, a total of 862 subjects were included in the meta‐analysis: 460 in the experimental group and 402 in the control group. Most of the study experimental groups allowed patients to bear weight within their tolerance levels as early as 2 weeks post‐operatively and asked them to perform active or active combined passive ankle movements [12, 15, 30, 31, 32, 33]. In the control group, the majority of patients were weight bearing at 6 weeks post‐operatively or chose not to bear weight, and ankle mobilisation was performed at 2 weeks [5, 12, 15, 27, 28, 30, 31, 32, 33]. In the study conducted by Yu Li et al. [5], the experimental group was bearing weight at the fourth week post‐operatively. In the study by Laarhovea et al. [29], the experimental group performed weight bearing on the fifth day post‐operatively and the form of ankle motion in the experimental and control groups was not reported. A study by Simanski et al. [28] reported that weight bearing was immediately initiated within the tolerated range following surgery. Smeeing et al. [2] reported that one experimental group performed weight bearing with protection on the 10th post‐operative day and that the other group performed unprotected weight bearing immediately after surgery; they did not report on the ankle mobility patterns of the experimental and control groups. In the research conducted by Park et al. [27], in addition to reporting the outcomes for all subjects during follow‐up, the results after the exclusion of subjects who did not perform the required weight bearing were reported separately, and only the latter data were included in the present study. Detailed information on the included studies is shown in Table 1.

**TABLE 1:** Information about the included studies.

Included studies	Age of subjects		Sex of subjects		Weight bearing start time		Forms of ankle movement		Outcome indicator
	Experimental group (M ± SD)	Control group (M ± SD)	Experimental group	Control group	Experimental group	Control group	Experimental group	Control group	
Qu Dai Biao et al., 2008 [33]	55.1 ± 12.7	55.8 ± 13.1	Males: 34	Males: 12	Post‐operative 2 weeks	Post‐operative 6 weeks	Proactive	‐‐	Olerud–Molander rating and time to return to work/life
			Females: 28	Females: 17					
Yang Chengfu et al., 2013 [32]	46.9 ± 7.8	45.2 ± 8.1	Males: 18	Males: 19	Post‐operative 2 weeks	Post‐operative 6 weeks	Proactive alliance passive activity	Proactive alliance passive activity	Baird–Jackson score
			Females: 7	Females: 6					
Li Lu Xue et al., 2020 [31]	47.4 ± 5.8	45.9 ± 4.3	Males: 15	Males: 17	Post‐operative 2 weeks	Post‐operative 6 weeks	Proactive	Proactive	AOFAS score and complication rate
			Females: 8	Females: 6					
AHI et al., 1993 [30]	55 (SD not reported)	55 (SD not reported)	Males: 4	Males: 3	Post‐operative 2 weeks	Non‐weight bearing	Proactive	Proactive	Olerud–Molander score and complication rate
			Females: 17	Females: 16					
Dehghan et al., 2016 [12]	41.7 ± 15.1	42.1 ± 15.4	Males: 32	Males: 27	Post‐operative 2 weeks	Post‐operative 6 weeks	Proactive	Proactive	Olerud–Molander score
			Females: 24	Females: 27					
Park et al., 2021 [27]	42.7 ± 14.2	43.1 ± 14.2	78 (gender not reported)	69 (gender not reported)	Post‐operative 2 weeks	Post‐operative 6 weeks	Proactive alliance passive activity	Proactive alliance passive activity	Olerud–Molander score, time to return to work/life and complication rate
Yu Li et al., 2021 [5]	39.18 ± 7.30	38.75 ± 8.33	Males: 25	Males: 22	Post‐operative 4 weeks	Post‐operative 13 weeks	Proactive	Proactive	AOFAS score
			Females: 19	Females: 20					
Schubert et al. 2020 [15]	46 ± 14	42 ± 16	Males: 13	Males: 16	Post‐operative 2 weeks	Post‐operative 6 weeks	Proactive alliance passive activity	Proactive alliance passive activity	Olerud–Molander score, complication rate
			Females: 12	Females: 9					
Laarhoven et al., 1996 [29]	35.5 (17–77)	37 (15–77)	Males: 24	Males: 21	Post‐operative 2 weeks	Post‐operative 6 weeks	‐‐	‐‐	Olerud–Molander score, time to return to work/life and complication rate
			Females: 17	Females: 19					
Smeeing et al., 2020 [2]	39.51 ± 14.60	37.8 ± 13.7	78 (gender not reported)	37 (gender not reported)	Post‐operative 5 days	Post‐operative 6 weeks	‐‐	‐‐	Olerud–Molander score, time to return to work/life and complication rate
Simanski et al., 2006 [28]	55.1 ± 12.7	55.8 ± 13.1	Males: 9	Males: 9	Immediate post‐operative weight bearing	Post‐operative 6 weeks	Proactive alliance passive activity	Proactive alliance passive activity	Olerud–Molander score and time to return to work/life, complication rate
			Females: 14	Females: 14					

### 3.3 Risk of bias evaluation

**FIGURE 2:**
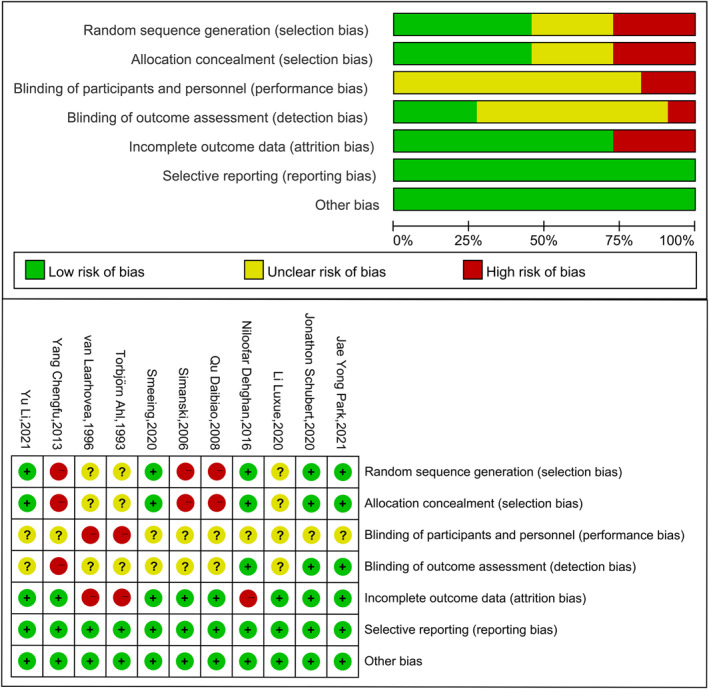
Overall evaluation of risk of bias of included studies.

Study risk of bias was evaluated using the Cochrane Collaboration's tool for assessing risk of bias in randomised trials (RoB1). Three of the studies [28, 32, 33] exhibited a high level of selection bias, all with nonrandom components in the generation of random sequences and where the experimenter might have anticipated the allocation results. Additionally, three studies [29, 30, 31] neglected to account for the randomisation and allocation concealment protocols. No studies were assessed as having a low risk of implementation bias, and no studies explicitly blinded the subjects, possibly because the intervention protocols were early weight bearing, which required informed consent from the subjects. One study [32] had a high level of detection bias, and seven studies [2, 5, 28, 29, 30, 31, 33] did not report whether they blinded the assessors. Three studies [12, 29, 30] had a high risk of attrition bias, mainly caused by the fact that the outcome indicator reported only the mean and not the standard deviation. All studies had a low risk of reporting bias and other biases. The overall risk of bias evaluation of the included studies is shown in Figure 2.

### 3.4 Results of the meta‐analysis of the effect of early weight bearing on functional recovery after ankle fracture surgery

#### 3.4.1 Ankle fracture recovery 6 weeks after surgery

**FIGURE 3:**
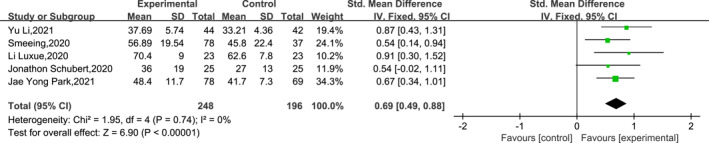
Ankle function 6 weeks post‐surgery.

A total of five papers reported the outcomes of two modalities of interventions for post‐operative ankle fracture function at 6 weeks post‐operatively. The outcome of the heterogeneity assessment suggested low variability among the studies (df = 4, *I* ^2^ = 0% and *p* = 0.74); thus, a fixed‐effects model was used for the meta‐analysis. Sensitivity analysis of the meta‐analysis results was performed using the leave‐one‐out method, and the results indicated that the results of the meta‐analysis were stable, with nonsignificant changes in heterogeneity (*I* ^2^ < 14%). Meta‐analysis results indicated that at 6 weeks post‐operatively, the ankle function scores were significantly higher in the EWB group than in the LWB group (SMD = 0.69, 95% CI: 0.49–0.88 and *p* < 0.001). The specific results are shown in Figure 3.

#### 3.4.2 Ankle fracture recovery 12 weeks after surgery

**FIGURE 4:**
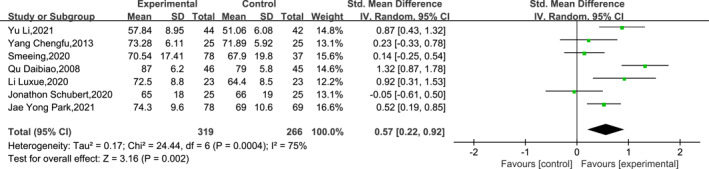
Ankle function 12 weeks post‐surgery.

A total of seven papers reported the outcomes of post‐operative functional recovery from ankle fractures at the 12th week post‐operatively. The heterogeneity analysis showed high heterogeneity (df = 6, *I* ^2^ = 75% and *p* = 0.0004), so a meta‐analysis was performed using a random‐effects model. Sensitivity analyses of the meta‐analysis results using the leave‐one‐out method showed insignificant changes in heterogeneity (*I* ^2^ > 59%) and more stable meta‐analysis results. The results of the meta‐analysis indicated that EWB was superior to LWB in terms of ankle function recovery at the 12th week post‐operatively (SMD = 0.57, 95% CI: 0.22–0.92, *p* = 0.002). The specific results are shown in Figure 4.

**FIGURE 5:**
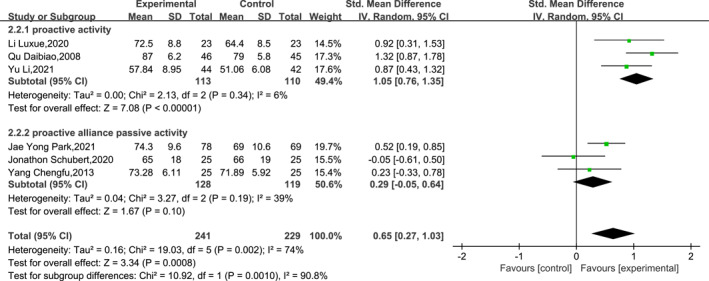
Subgroup analysis of ankle function 12 weeks post‐surgery.

Subgroups were analysed according to the type of post‐operative ankle movement (active vs. active combined passive movement) in the experimental group. Notably, due to the absence of reported ankle activity details in Smeeing et al.'s [2] study, it was consequently excluded from the subgroup analysis. The heterogeneity analysis indicated low heterogeneity, so the subgroups were analysed using a fixed‐effects model. The results of the subgroup analysis showed a significant effect of the form of ankle motion on ankle function recovery at the 12th week post‐operatively (df = 1, *I* ^2^ = 90.08%, *p* = 0.001). The specific results are shown in Figure 5.

#### 3.4.3 Ankle fracture recovery at 24–26 weeks post‐operatively

**FIGURE 6:**
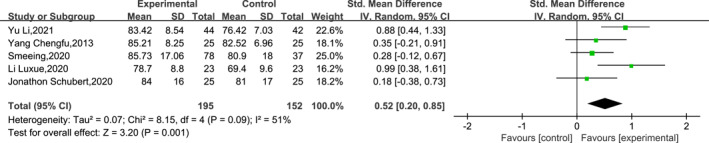
Ankle function 24–26 weeks post‐surgery.

A total of five papers reported on the recovery of ankle function at 24–26 weeks post‐operatively. The results of the heterogeneity test indicated low heterogeneity among the included studies (df = 4, *I* ^2^ = 51%, *p* = 0.09), so a meta‐analysis was performed using a random‐effects model. The results of the sensitivity analysis via the leave‐one‐out method indicated an *I* ^2^ ranging from 34% to 61%. The results of the meta‐analysis indicated that EWB was significantly more effective than LWB in improving post‐operative functional recovery of the ankle at 24–26 weeks post‐operatively (SMD = 0.52, 95% CI: 0.20–0.85 and *p* = 0.001). The specific results are shown in Figure 6.

**FIGURE 7:**
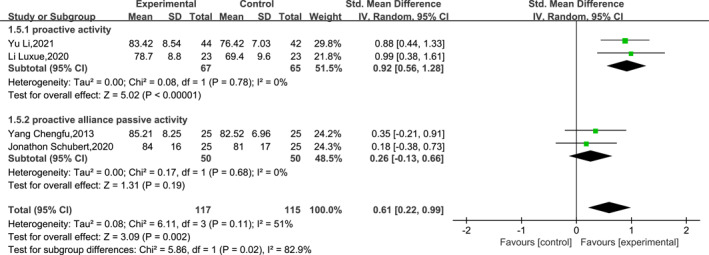
Subgroup analysis of ankle function 24–26 weeks post‐surgery.

Subgroups were analysed according to the type of post‐operative ankle movement (active vs. active combined passive movement) in the experimental group. Due to the absence of reported ankle activity details in Smeeing et al.'s [2] study, it was consequently excluded from the subgroup analysis. The heterogeneity analysis indicated low heterogeneity, so the subgroups were analysed using a fixed‐effects model. The results of the subgroup analysis showed a significant effect of the form of ankle motion on ankle function recovery at the 12th week post‐operatively (df = 1, *I* ^2^ = 82.9% and *p* = 0.02). The specific results are shown in Figure 7.

#### 3.4.4 One year after ankle fracture surgery

**FIGURE 8:**
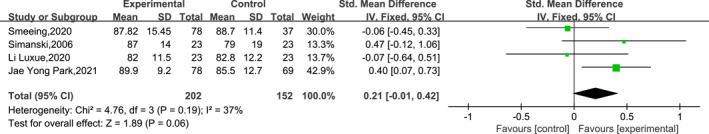
Ankle function 1 year post‐surgery.

A total of four papers were reported on the recovery of ankle function at the first year post‐operatively. The results of the heterogeneity analysis indicated low heterogeneity among the included studies (df = 3, *I* ^2^ = 37% and *p* = 0.19), so a meta‐analysis was performed using a fixed‐effects model. Sensitivity analyses were performed using the leave‐one‐out method, and the results indicated that the meta‐analysis results were stable (*I* ^2^ < 48%). The results of the meta‐analysis indicated that there was no difference in the recovery of ankle function between the EWB group and the LWB group at the first year post‐operatively (SMD = 0.21, 95% CI: −0.01–0.42 and *p* = 0.06). The specific results are shown in Figure 8.

### 3.5 Results of the meta‐analysis of the time to return to work/daily life

**FIGURE 9:**
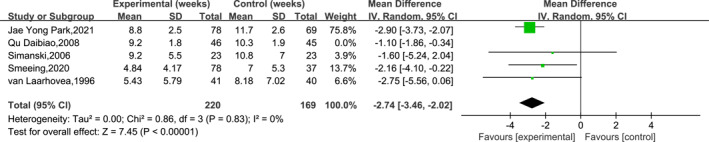
Meta‐analysis results of the time to return to work/life.

A total of five papers were reported at the time for patients to return to work/life, and the units were weeks. The results of the heterogeneity analysis indicated a high level of heterogeneity among the included studies (df = 4, *I* ^2^ = 61% and *p* = 0.04), so a meta‐analysis was performed using a random‐effects model. Sensitivity analyses using the leave‐one‐out method showed a significant reduction in the heterogeneity after the exclusion of 1 study, and the results of the meta‐analysis were highly stable (*I* ^2^ = 0%). The subjects in the study by Qu Daibiao et al. [33] were concurrently given Chinese herbal medicines post‐operatively, and the EWB group was allowed to undergo pressure stimulation on the ankle joint as early as 1 week post‐operatively, which may have had some impact on the patients' time to return to work/life. The meta‐analysis results showed that the EWB group returned to work/daily life earlier than the LWB group (MD = −2.74, 95% CI:−3.46 to −2.02 and *p* < 0.001). The specific results are shown in Figure 9.

### 3.6 Results of the meta‐analysis of complication rates

**FIGURE 10:**
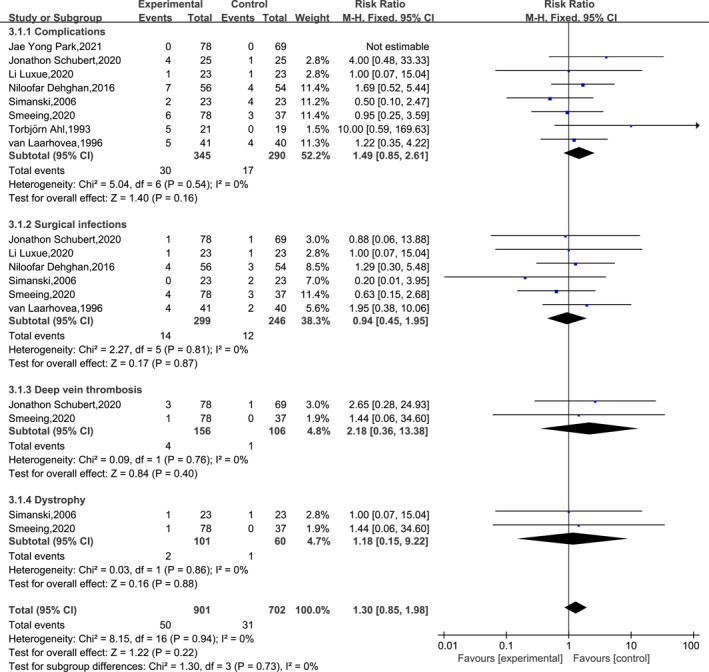
Results of the meta‐analysis of complication rates.

A total of eight papers reported the number of patients with complications in each group. Detailed information on complications is shown in Table 2. The results of the heterogeneity test indicated low heterogeneity among the included studies (df = 6, *I* ^2^ = 0% and *p* = 0.54), so a meta‐analysis was performed using a fixed‐effects model. Sensitivity analyses were performed using the leave‐one‐out method, and the results indicated that the meta‐analysis results were stable (*I* ^2^ = 0%). The meta‐analysis results indicated that there was no significant difference in the complication rate between the EWB and LWB groups (RR = 1.49, 95% CI: 0.85–2.61 and *p* = 0.16). In addition, meta‐analysis of different types of complications indicated that there was no significant difference in the incidence of superficial infections (RR = 0.94, 95% CI: 0.45–1.95 and *p* = 0.87), deep vein thrombosis (RR = 2.18, 95% CI: 0.36–13.38 and *p* = 0.40) and dystrophy (RR = 1.18, 95% CI: 0.15–9.22 and *p* = 0.88) in EWB versus LWB. The specific results are shown in Figure 10 and Table 2.

**TABLE 2:** Complications details.

Complications	Rate of incidence	Severity	Description	Time of appearance
Li Lu Xue et al., 2020 [31]	Experimental group: 1/23	Unlabelled	Surgical infections (EWB 1, LWB 1)	——
	Control group: 1/23			
AHI et al., 1993 [30]	Experimental group: 5/21	Unlabelled	——	——
	Control group: 0/19			
Dehghan et al., 2016 [12]	Experimental group: 7/56	Unlabelled	Superficial infections (EWB 4 and LWB 3)	Six weeks post‐operative
	Control group: 4/54		Wound healing complications (EWB 3 and LWB 1)	
Park et al., 2021 [27]	Experimental group: 0/78	——	——	——
	Control group: 0/69			
Schubert et al., 2020 [15]	Experimental group: 4/78	Unlabelled	Deep vein thrombosis (EWB 3 and LWB 1)	Six weeks post‐operative (1 DVT)
	Control group: 1/69		Superficial infection (EWB 1 and LWB 1)	Twelve weeks post‐operative (3 DVT1 superficial infection)
Laarhoven et al., 1996 [29]	Experimental group: 5/41	Unlabelled	Superficial wound infections (EWB 4 and LWB 2)	——
			Osteitis (EWB 0 and LWB 1)	
	Control group: 4/40			
			Wound dehiscence (EWB 1 and LWB 1)	
Smeeing et al., 2020 [2]	Experimental group: 6/78	Unlabelled	Superficial wound infections (EWB 4, LWB 3)	——
			Dystrophy (EWB 1 and LWB 0)	
	Control group: 3/42		Deep venous thrombosis (EWB 1 and LWB 0)	
Simanski et al., 2006 [28]	Experimental group: 2/23	Unlabelled	Dystrophy (EWB 1 and LWB 1)	——
			Allergic reaction (EWB 1 and LWB 0)	
			Pseudarthrosis of the fibula (EWB 0 and LWB 1)	
	Control group: 4/23			
			Superficial wound infections (EWB 0 and LWB 2)	

## 4 DISCUSSION

This study found that patients who received post‐operative EWB interventions had significantly better recovery than those who received post‐operative LWB interventions in terms of ankle function scores. In contrast, there was no statistically significant difference between the effects of EWB and LWB on ankle function scores at 1 year post‐operatively. In addition, EWB allowed patients to return to work/daily life earlier, and the difference in the complication rate between EWB and LWB groups was not statistically significant.

The Olerud–Molander score assesses ankle function in nine areas: pain, ankle mobility, swelling, stair climbing, running, jumping, squatting, walking and support. The AOFAS score assesses ankle function in terms of pain, function (including activity limitations, maximum walking distance, walking surface, gait abnormality, sagittal motion, hindfoot motion and ankle hindfoot stability) and alignment. The Baird–Jackson score evaluates ankle function in terms of pain, stability of ankle, able to walk, able to run and ability to work, Movements of ankle and radiological result showed that all three scales range from 0 to 100. We therefore combined the results of these three scales for meta‐analysis. These scale scores reflect the ankle in a consistent way, where a higher score is associated with better ankle function.

All studies supported that EWB helped improve ankle function at the sixth post‐operative week, and the heterogeneity among the studies was low. This is consistent with the results of previously published meta‐analyses [20, 21, 34]. Results of these indicated that EWB was effective in improving ankle function in the early stages of rehabilitation compared to LWB. Additionally, Dehghan et al. [12] reported more detailed functional ankle outcomes in their study, stating that at 6 weeks post‐operatively, there was a significant improvement in ankle mobility in patients who underwent EWB compared to those who underwent LWB. However, EWB does not seem to be effective in reducing pain in post‐operative ankle fracture patients. Yang et al. [32] and Schubert et al. [15] have both reported in their studies that there was no difference in subjective pain perceived by patients undergoing EWB and LWB at 6 weeks post‐operatively. There are physiologically relevant studies that also support the role of early weight bearing in accelerating recovery after fracture surgery. In particular, Eric et al. [35] conducted a biomechanical simulation of early weight bearing after ankle fracture surgery and found minimal fracture displacement in patients with weight bearing, which indicated that early weight bearing after ankle fracture surgery is biomechanically feasible and safe, with a lower risk of dislocation or fixation failure. Notably, animal studies by Gardner et al. [36] demonstrated that mechanical stress stimulation administered after fracture surgery contributes to bone scab formation. Similarly, Jian‐Huang et al. [37] showed that mechanical forces acting on skeletal tissues can remodel and alter their structure, stimulating differentiation, maturation and apoptosis of osteoblasts and osteoclasts.

At the 12th week post‐operatively and at the 24th–26th week post‐operatively, this meta‐analysis showed that ankle function scores were higher in patients with EWB, a result that differs from some published meta‐analyses. For instance, Sharma's meta‐analysis [20] indicated that at the 10th–12th week post‐operatively, patients with EWB had better ankle function scores than those with LWB, and this result is similar to that of the present study. Nevertheless, after 6 months post‐operatively, there was no significant difference in the recovery of ankle function between the EWB and LWB groups. However, the results of a meta‐analysis by Khojaly et al. [21] indicated that there was no significant difference in the ankle function scores of patients with EWB at the third month post‐operatively compared to those with LWB. The reasons for the discrepancy may be related to the inconsistent delineation of post‐operative time phases and differences in the included studies. Sharma et al. only analysed the results of the last assessment at 6 months after surgery, whereas in the present study, only the functional scores at the 24^th^–26^th^ week were selected for analysis. Furthermore, unlike Khojaly et al.'s study, the studies included in this study reported more ankle function scores at the 12th week post‐operatively. Most of these studies were published after the retrieval date of the study by Khojaly et al., and the results were mostly in favour of EWB. Additionally, the randomised controlled studies that were not included in the meta‐analysis due to lack of standard deviation also indicated that the EWB group had higher ankle function scores at the 12th week post‐operatively [12, 29, 30].

A subgroup analysis of the results at the 12th post‐operative week based on the form of ankle motion was performed in this study to identify sources of heterogeneity across studies at post‐operative week 12. Heterogeneity was reduced within the subgroups of active movement as well as active combined with passive movement, and significant differences were demonstrated among the subgroups. Subgroup analyses showed that at the 12th post‐operative week, ankle function scores were better in patients undergoing EWB along with active movement than in those who performed active combined with passive movement. This may be related to the fact that passive activities are more likely to exceed the range of motion that the joints can tolerate. Some studies have shown an increased risk of wound nonhealing and infection with post‐operative movement beyond joint limitations [34], and unfavourable factors that lead to an increased risk of complications may also lead to oedema and pain in the joints. Most of the studies included in this meta‐analysis evaluated ankle function using the Olerud–Molander scale and the AOFAS scale, which include at least one evaluation of ankle oedema and pain, and it is possible that passive movement may influence the evaluation of ankle function by both patients and healthcare professionals. More high‐quality randomized controlled trials are still needed to validate this idea. In particular, there is also a need for more high‐quality evidence on the need for ankle mobilisation in patients with EWB. Honigmann et al. [38], who observed the effect of ankle mobility on ankle function scores in patients with EWB, found no difference in ankle function scores between the experimental and control groups in the first 10 weeks. Therefore, with limited evidence, EWB combined with active movement appears to be a more favourable option for the post‐operative rehabilitation of patients with ankle fractures.

No differences in ankle function at the first year post‐operatively in patients who underwent EWB compared with LWB were shown in the results of this meta‐analysis. This indicates that EWB will not cause a decrease in ankle function in the later stages of a patient's post‐operative rehabilitation and that it is no less effective than LWB for the post‐operative rehabilitation of patients with ankle fractures. This is supported by a number of retrospective analyses that were not included in this study [7, 39]. It is considered that EWB brings about no difference in post‐operative ankle functional outcomes compared to conventional LWB.

In addition to differences in ankle function scores, differences between the EWB and LWB groups were also seen in the time it took for patients to return to work/daily life. The results of the meta‐analysis indicated that patients with EWB were able to return to work/daily life earlier, which is more convenient for the patients and could potentially reduce their financial stress. A retrospective study conducted by Cunningham et al. [11] also pointed out that EWB significantly accelerated the time to return to work for physically active patients. Notably, a prospective study by Peter et al. [18] statistically estimated the economic losses that would be borne by ankle fracture patients in post‐operative rehabilitation, most of which stemmed from loss of income. Returning to work earlier may help reduce the costs associated with the injury for ankle fracture patients. Nonetheless, some studies have also concluded that there is no difference in the time to return to work between the EWB and LWB groups [40]. Given that there are also various influencing factors on the return‐to‐work process, more research is still needed to determine whether EWB can provide financial benefits to post‐operative ankle fracture patients.

Complications are also an aspect that needs to be focused on. The results of the meta‐analysis showed no statistically significant difference in the complication rate between patients with EWB and LWB, which is consistent with the results of a previously published study on this subject [21, 34]. Notably, a multicentre retrospective study conducted by Bando et al. [39] further revealed differences between the types of complications occurring in the EWB and LWB groups and showed that there were no differences between the two groups in terms of skin surface infections, fracture nonhealing, skin blisters, neurological disuse and nonhealing of surgical wounds and that deep tissue infections and secondary surgeries were less prevalent in the EWB group than in the LWB group. Thus, there is significant evidence to suggest that early weight bearing after ankle surgery has a safety profile at least comparable to conventional LWB.

Several limitations also exist in this study. Firstly, this study used ankle function rating scales as the primary indicators, among which however, the total scores of these scales cannot reflect more specific ankle functions (e.g., joint morphology, joint mobility and bracing time), and therefore, which specific ankle functions are affected by EWB need to be further investigated. Secondly, there were three studies included in this study that evaluated ankle function with the AOFAS scale, which has been reported to be unreliable with limited precision, interobserver variability, non‐informative conclusion, etc. Therefore, the results of the included AOFAS scales have the potential to influence the reliability of the results of this study. Lastly, some of the studies included in this study were at higher risk of bias, particularly performance bias. Although it was difficult to blind the participants in this study due to the experimental design, it was necessary to blind them because of the more subjective content of the outcome indicators. There were some studies with a high risk of detection bias, which may have some impact on the quality of the results of this study.

## 5 CONCLUSION

In summary, EWB was found to be effective in improving ankle function in post‐operative ankle fracture patients, and it is possible that the type of ankle movement may have an effect on the effectiveness of EWB. Patients who receive EWB interventions do not have lower functional ankle outcomes than those who receive LWB interventions and are able to return to work/daily life earlier. The EWB rehabilitation protocol also resulted in a complication rate no higher than that of the traditional LWB protocol, providing a high degree of safety.

## AUTHOR CONTRIBUTIONS

**Bocheng Chen**: Writing – original draft preparation, data curation. **Ziyan Ye**: Data curation. **Jiaxin Wu**: Validation, data curation. **Tiancheng Yu**: Validation, writing – review & editing. **Guoxiang Wang**: Conceptualization, project administration.

## CONFLICT OF INTEREST STATEMENT

The author(s) declared no potential conflicts of interest with respect to the research, authorship and/or publication of this article. We wish to assert that there is no conflict of interest associated with any of the authors or organisations involved in this study. We declare that we have no financial or personal relationships that could influence the findings or interpretation presented in this paper.

## ETHICS STATEMENT

Not applicable.

## CONSENT FOR PUBLICATION

We agree for publication.

## Supporting information

Supporting Information S1

## Data Availability

The data sets used or analysed during the current study are available from the corresponding author on reasonable request.
